# Multicountry Burden of Chronic Hepatitis C Viral Infection among Those Aware of Their Diagnosis: A Patient Survey

**DOI:** 10.1371/journal.pone.0086070

**Published:** 2014-01-21

**Authors:** Marco daCosta DiBonaventura, Yong Yuan, Benedicte Lescrauwaet, Gilbert L’Italien, Gordon G. Liu, Isao Kamae, Josephine A. Mauskopf

**Affiliations:** 1 Health Outcomes Practice, Kantar Health, New York, New York, United States of America; 2 Global Health Economics and Outcomes Research, Bristol-Myers Squibb, Princeton, New Jersey, United States of America; 3 Xintera Consulting, Brussels, Belgium; 4 Yale University School of Medicine, New Haven, Connecticut, United States of America; 5 National School of Development, Peking University, Beijing, China; 6 Graduate School of Public Policy, The University of Tokyo, Tokyo, Japan; 7 RTI International, Research Triangle Park, North Carolina, United States of America; Centers for Disease Control and Prevention, United States of America

## Abstract

**Background:**

The World Health Organization has called for global and regional assessments of the burden of hepatitis C (HCV) along with country-specific patient profiles to better inform healthcare policy. The present investigated the characteristics and burden of patients reporting a diagnosis of HCV infection in the US, France, Germany, Italy, Spain, the UK, urban China, and Japan using a consistent methodology of patient-reported surveys.

**Methods:**

The 2010 5EU (N = 57,805), 2009 US (N = 75,000), 2008/2009 Japan (N = 37,683), and 2009/2010 urban China (N = 33,261) waves of the National Health and Wellness Survey were used as the data source. Within each country, patients with a self-reported diagnosis of HCV were compared with those who did not report a diagnosis of HCV on sociodemographics, health behaviors, comorbidities, and health outcomes (e.g., Short Form-12v2). The effect of HCV was examined using regression analysis applying sampling weights.

**Results:**

The prevalence of HCV ranged from 0.26% (China) to 1.42% (Italy). Patients in Japan and Italy (61.60 and 61.02 years, respectively) were the oldest, while patients in the US were the most likely to be obese (39.31%) and have concomitant anxiety (38.43%) and depression (46.05%) compared with other countries. Pooling countries and adjusting for sociodemographics, health behaviors, and comorbidities, HCV was associated with significantly lower physical component summary scores (b = −2.51) and health utilities (b = −0.04) and greater overall work impairment (b = 8.79), physician visits (b = 2.91), and emergency department visits (b = 0.30) (all p<.05). The effects on health status were strongest in the US and UK while the effects on healthcare resource use were strongest in Japan.

**Conclusions:**

HCV was associated with a significant humanistic and economic burden. These results suggest that the manifestation of the HCV burden, and the profile of the patients themselves, varied dramatically by country. Successful disease management should be cognizant of region-specific unmet needs.

## Introduction

Approximately 85% of patients who are infected with the hepatitis C virus (HCV) develop chronic HCV infection [1]. Although often initially asymptomatic, patients with chronic HCV infection are at a significantly higher risk for cirrhosis, hepatocellular carcinoma (HCC), and liver transplantation in later years [2]. Indeed, HCV infection is the most common reason for liver transplantation and accounts for over 33% of HCC cases in the United States (US) [3].

Recent systematic reviews and meta-analyses have documented the detrimental effect that chronic HCV infection has on the health status of patients across a number of countries [4]–[6]. In the US, DiBonaventura et al (2010) reported moderately-sized effects between patients with HCV and matched controls with respect to physical component summary scores of the Short Form-12, an instrument used to assess health status/health-related quality of life (39.57 vs. 42.66, respectively) [7]. Although fewer in number, studies outside the US have drawn similar conclusions. In some studies, such as those conducted in Germany, Italy, and Japan, health status scores from patients with HCV have been compared with population norms, highlighting the burden associated with infection [8]–[10]. Häuser et al (2004), for example, showed that patients with chronic HCV in Germany reported mean physical and mental summary scores (from the Short Form-36, a longer form of the Short Form-12 described above) of 40.94 and 43.21, respectively, both lower than the population mean of 50 [8]. In other studies, such as those in Spain, the United Kingdom (UK), and China, health status scores were compared between patients with HCV and non-HCV controls, also providing support for the deleterious effect HCV can have on mental and physical functioning [11]–[13].

HCV infection has also been found to be associated with increased healthcare resource utilization though, as with health status, this has been predominantly supported by US-focused studies [7], [14]. One study found that patients with HCV infection reported 34% more emergency room (ER) visits, and 30% more physician visits in the prior six months [7]. Similarly, using patient-reported surveys in both Japan and Europe, patients who reported a diagnosis of HCV reported a significantly higher number of ER visits, hospitalizations, and physician visits, compared with propensity-score matched cohorts [15]–[16].

Indirect costs have also been found to be significantly higher among patients with HCV. Analyzing data from an employer database, Su et al (2010) found those infected with HCV reported significantly higher rates of absenteeism (i.e., health-related work absences) and short- and long-term disability [17]. A study using data from the US National Health and Wellness Survey found significantly less labor force participation overall among patients with and HCV and, among those in the labor force, higher rates of both absenteeism and presenteeism (i.e., health-related decreases in work productivity while at work) compared with a matched control group [18]. Similarly, Liu et al (2012) also found that absenteeism and overall work impairment were higher among patients in Japan relative to matched controls [15].

Although many of the above studies have highlighted the burden of HCV across a range of outcomes, these studies are often limited to the US. Even in cases where studies in different countries included the same outcome (e.g., health status), cross-country comparisons are difficult to make. Differences observed between studies in different countries may be a reflection of the specific needs of those patients based on their unique characteristics and healthcare systems. On the other hand, differences observed between studies in different countries may merely be the result of methodological differences in data collection.

The varied methods used in current studies to estimate the burden of HCV infection in different countries indicates the value for a more standardized multicountry assessment of patients with HCV. Indeed, the World Health Organization has called for global and regional assessments of the burden of HCV along with additional country-specific profiles of patients to better inform policy and action [19]. Currently, many countries are ill-equipped for HCV surveillance and are in a poor position to make policy decisions based on adequate data [19]. As a result, the aim of the present study was to investigate the patient characteristics and burden of patients reporting a diagnosis of HCV infection in the US, France, Germany, Italy, Spain, the UK, urban China, and Japan using a consistent methodology of population-based, patient-reported surveys. Because each country is unique with respect to its epidemiological history of HCV, using a standardized methodology can help to identify differences in the patient experience across countries to inform proper disease management.

## Methods

### Data Source: National Health and Wellness Survey

The current study used data from the 2010 5EU (N = 57,805), 2009 US (N = 75,000), 2008/2009 Japan (N = 37,683), and 2009/2010 urban China (N = 33,261) waves of the National Health and Wellness Survey (NHWS). Prior publications have outlined the methodology for each of the countries [7], [15]–[16], [20]. Briefly, the NHWS is an annual cross-sectional population-based health survey of adults (18 years and older) fielded in each of the countries mentioned above. In most cases, respondents were recruited from a large Internet panel, with the survey being completed online. In places with poor internet penetration (parts of Italy, Spain, and urban China), offline recruiting was also conducted. Offline recruitment consisted of respondents being recruited to a centralized facility to complete the survey instrument. In each country, potential respondents (online or offline) were recruited to mimic the demographic distributions of the adult population in that country based on available government statistics provided by the International Database of the United States Census [21]. More specifically, the proportions by each age and sex stratum (e.g., 18–29 year old males) were identified from these governmental statistics and then mirrored during the recruitment of NHWS respondents.

### Ethics Statement

All respondents provided informed consent electronically prior to answering any survey questions. Because the survey was administered entirely online, written consent was not possible. All electronic forms of consent were saved and stored associated with each respondent’s unique identifier. All respondents were only known by a unique identifier. The survey and procedure was approved by an Institutional Review Board (Essex Institutional Review Board, Lebanon, NJ).

### Measures

#### HCV diagnosis

Because the NHWS is patient-reported, only awareness of an HCV diagnosis was assessed (as opposed to an assessment of clinical records or HCV antibodies). NHWS respondents are presented with a list of conditions that they had ever been diagnosed with, one of which was HCV. Respondents who reported ‘yes’ were included in the HCV group and all others were included in the control group. Although acute versus chronic infection was not assessed, the clear majority of patients would have chronic HCV infection [1].

#### Demographics

Age, marital status (married/living with partner or all else), annual household income (categorized as below the country-specific median, above the country-specific median, or decline to answer to allow for a consistent assessment across countries), and employment status (currently employed or not currently employed) were assessed for all respondents.

#### Health behaviors

The NHWS also captures additional information on each respondent’s health characteristics including their height and weight (the latter can be left unanswered). Height and weight were then used to calculate body mass index (BMI) and associated BMI categories: underweight, normal weight, overweight, obese, or decline to answer (the category reserved for those who did not provide their weight). Smoking status (current smoker versus non-smoker), alcohol use (at least one alcoholic drink per month versus abstaining from alcohol), and exercise behavior (at least one day in the past month of vigorous exercise versus no exercise) were also assessed.

#### Comorbidities

Based on the list of conditions presented to all respondents in the NHWS, the Charlson comorbidity index (CCI) score was then calculated to provide a measure of overall comorbidity burden [22]. Each condition in the index is given an assigned weight based on its relationship with future mortality and the weights are then summed to create a total index score (the higher the score the greater the comorbidity burden). As the CCI does not include psychiatric conditions, reported diagnosis of anxiety and depression were also included as additional comorbidities.

#### Health outcomes

Health status was measured using the Short-Form 12 version 2 (SF-12v2), a twelve-item instrument which has extensive evidence for its reliability and validity [23]. The twelve items of the SF-12v2 are used in a scoring algorithm to produce both a mental (MCS) and physical component summary (PCS). These scores have a population mean and standard deviation of 50 and 10, respectively, and represent the level of mental and physical health of each respondent (higher scores represent better health). Similarly, the items of the SF-12v2 are also used to create a health utility score, which were derived using the SF-6D algorithm [23]. Common in economic valuations of health, health utility scores vary from 0 (representing a health state similar to death) to 1 (representing a health state of perfect health). Work productivity was measured using the Work Productivity and Activity Impairment questionnaire, which included measures of absenteeism (defined here as the percentage of work time missed due to health), presenteeism (defined here as the percentage of health-related impairment experienced while at work), overall work impairment (the total percentage of work time that was missed due to either absenteeism or presenteeism), and activity impairment (the percentage of impairment experienced during daily activities) [24]. Self-reported healthcare resource utilization included the number of provider visits, emergency room visits, and hospitalizations in the past six months.

### Statistical Analyses

Within each country, comparisons between those with and without a diagnosis of HCV were made with chi-square tests and independent-samples t-tests for demographic and health characteristic variables. These analyses applied country-specific sampling weights to project to the population. For ease of presentation, only the weighted percentages and weighted means for those with HCV in each country are reported. Although the percentages and means which significantly differ between those with and without an HCV diagnosis are noted, the values for the non-HCV group are not shown. They are available upon request from the authors.

Pooling all countries together, the effect of an HCV diagnosis on health outcomes was tested using linear regression, applying sampling weights to project to the country-level population. All models controlled for country, age, gender, marital status, annual household income, BMI, alcohol use, smoking status, and the CCI. These covariates were selected as they have been found to be significant predictor of outcomes in other HCV studies [7], [15]–[16], [18]. Country-specific models were also conducted using the same modeling framework and covariates (excluding country). Adjusted means are reported. These were calculated by setting the covariates at mean level across all countries.

## Results

The projected prevalence of an HCV diagnosis across each country is as follows: United States (1.08%), France (0.59%), Germany (0.44%), the UK (0.35%), Italy (1.42%), Spain (0.82%), China (0.26%), and Japan (0.75%).

Substantial variability was also observed across the countries with respect to the characteristics of respondents with an HCV diagnosis (see **Table 1**). Although more than half of respondents who reported a diagnosis of HCV were male in each country (Italy: 54.76% male to Spain: 67.39% male), the pattern was reversed in France, where only 36.03% of respondents who reported a diagnosis were male. Between 48.93% and 64.26% of respondents reporting a diagnosis of HCV reported household incomes below the respective country median (except for urban China where 23.82% reported incomes below the median). Several differences were observed with respect to age, highlighting the differences in the historical epidemiology. Respondents in Italy (Mean = 61.02) and Japan (Mean = 61.60) were the oldest while those in urban China were the youngest (Mean = 39.11). The mean age of respondents in the remaining countries varied between 45.98 years (Spain) and 56.13 years (France). These differences across countries are slightly less extreme when taking into account the ages of the respondents who did not report a diagnosis of HCV within each country. For example, Italy and Japan have the oldest overall adult populations and urban China with the youngest.

**Table 1:** Demographic and health history data for respondents who reported a diagnosis of HCV in each country.

	US		France		Germany		UK		Italy		Spain		China (urban)		Japan	
	HCV (n = 808)	No HCV	HCV (n = 78)	No HCV	HCV (n = 65)	No HCV	HCV (n = 52)	No HCV	HCV (n = 97)	No HCV	HCV (n = 44)	No HCV	HCV (n = 91)	No HCV	HCV (n = 312)	No HCV
Male	**60.46%** *	48.13%	**36.03%** *	48.30%	**56.11%**	48.70%	**61.66%**	49.05%	**54.76%**	48.60%	**67.39%** *	48.32%	**61.26%**	50.64%	**54.90%**	48.82%
Married/living with partner	**52.05%** *	59.30%	**49.97%** *	64.58%	**37.91%** *	60.32%	**64.23%**	62.75%	**66.25%**	64.20%	**68.03%**	61.55%	**76.15%**	79.29%	**74.03%**	66.09%
Household income: Below median	**64.26%** *	48.84%	**58.32%**	52.26%	**54.15%** *	39.99%	**53.75%**	45.99%	**48.93%**	41.70%	**55.23%**	50.02%	**23.82%** *	38.61%	**54.48%** *	42.31%
Household income: Above median	**32.76%** *	45.22%	**29.84%**	36.19%	**36.59%**	42.64%	**40.71%**	42.14%	**40.2%**	41.49%	**37.98%**	34.48%	**76.18%** *	57.96%	**39.43%**	48.08%
Household income: Decline to answer	**2.98%** *	5.94%	**11.84%**	11.54%	**9.26%** *	17.37%	**5.54%**	11.87%	**10.88%**	16.81%	**6.79%** *	15.50%	**0.00%**	3.42%	**6.09%**	9.61%
BMI: Underweight	**0.72%** *	1.89%	**5.07%**	3.24%	**1.02%**	1.98%	**10.23%**	2.25%	**0.00%**	3.01%	**2.16%**	1.99%	**12.62%**	8.66%	**11.53%**	10.22%
BMI: Normal weight	**26.82%** *	30.82%	**38.32%**	47.49%	**37.2%**	38.50%	**26.05%**	35.67%	**49.53%**	48.42%	**44.98%**	42.81%	**68.25%**	65.65%	**73.25%**	69.06%
BMI: Overweight	**32.66%**	32.09%	**42.02%**	31.77%	**40.76%**	35.32%	**44%**	33.10%	**30.21%**	33.58%	**39.54%**	37.48%	**13.31%**	17.28%	**12.62%**	15.30%
BMI: Obese	**39.31%** *	33.26%	**14.59%**	15.98%	**21.02%**	21.88%	**16.48%**	23.60%	**20.26%**	13.60%	**13.33%**	16.68%	**5.83%**	6.72%	**1.9%**	2.57%
BMI: Missing	**0.49%** *	1.94%	**0.00%**	1.53%	**0.00%**	2.32%	**3.25%**	5.38%	**0.00%**	1.38%	**0.00%**	1.03%	**0.00%**	1.70%	**0.7%** *	2.85%
Currently exercise	**52%** *	64.37%	**48.32%**	54.16%	**56.47%**	58.42%	**68.47%**	59.64%	**54.2%**	54.31%	**49.29%**	57.35%	**59.83%**	50.48%	**51.3%**	47.83%
Currently drink	**56.57%** *	65.41%	**67.99%**	78.82%	**79.87%**	81.00%	**80.46%**	83.61%	**63.11%**	67.30%	**59.47%**	69.90%	**81.4%** *	59.20%	**57.72%** *	73.04%
Currently smoke	**55.04%** *	22.68%	**45.12%** *	29.20%	**55.12%** *	31.01%	**53.5%** *	21.81%	**39.81%** *	25.08%	**52.54%** *	29.48%	**49.91%** *	22.68%	**24.67%**	24.86%
Diagnosed anxiety	**38.43%** *	16.18%	**30.88%** *	18.36%	**25.31%** *	7.40%	**36.16%** *	15.89%	**23.66%**	14.67%	**31.78%** *	16.62%	**17.65%** *	3.57%	**4.17%**	4.90%
Diagnosed depression	**46.05%** *	18.00%	**24.05%** *	7.87%	**39.46%** *	9.23%	**31.42%** *	16.67%	**7.40%**	7.38%	**29.62%** *	9.58%	**5.91%**	2.16%	**6.12%**	4.75%
Currently employed	**43.73%** *	55.97%	**26.22%** *	49.89%	**51.58%**	56.05%	**59.89%**	55.32%	**38.31%** *	54.35%	**60.11%**	49.64%	**92.53%** *	68.55%	**42.69%** *	57.61%
Currently in the labor force	**53.04%** *	64.54%	**34.79%** *	57.19%	**54.64%**	60.85%	**64.93%**	60.23%	**40.48%** *	58.80%	**69.57%**	60.48%	**94.13%** *	69.95%	**44.03%** *	60.34%
Age (Mean)	**49.89** *	45.98	**56.13** *	47.27	**52.49** *	48.38	**45.58**	46.91	**61.02** *	50.61	**45.98**	49.88	**39.11** *	43.66	**61.60** *	48.18
CCI (Mean)	**1.55** *	0.40	**0.92** *	0.23	**0.85** *	0.33	**2.56** *	0.27	**0.83** *	0.26	**1.61** *	0.34	**1.11** *	0.24	**1.97** *	0.32

CCI = Charlson comorbidity index.

p<.05 when compared with respondents without HCV within the same country.

Despite the clear elevated risk for liver-related sequelae, the majority of respondents reporting a diagnosis of HCV continued to regularly drink alcohol, particularly in Germany (79.87%), the UK (80.46%), and urban China (81.40%). In most countries, rates of alcohol use were no different than respondents without HCV. The exceptions to this were the US and Japan, where respondents with HCV had significantly lower rates than the non-HCV respondents, and urban China, where respondents with HCV had significantly higher rates than non-HCV respondents.

Large differences were observed with respect to overall comorbidity burden. All respondents with HCV reported a significantly greater comorbidity burden than their non-HCV counterparts. Respondents in the UK and Japan reported the greatest burden (Mean CCI = 2.56 and 1.97, respectively) which is even more extreme when viewed in relation to their non-HCV counterparts in those countries (Mean CCI = 0.27 and 0.32, respectively). Although in most countries respondents with HCV reported a CCI score approximately 3 times greater than non-HCV controls, respondents with HCV in Italy and Japan reported CCI scores approximately 9 and 6 times higher. On an absolute level, respondents with HCV in urban China reported a modest burden (Mean CCI = 1.11), though this value was nearly 5 times greater than non-HCV respondents in urban China.

With the exception of Italy and Japan, levels of anxiety and depression were significantly higher among respondents with HCV compared with respondents without HCV in each country. In most cases, these conditions were approximately two to three times as frequent among respondents with HCV though depression was approximately four times more likely in France (24.05% vs. 7.87%) and Germany (39.46% vs. 9.23%) and anxiety was approximately four times more likely in urban China (17.65% vs. 3.57%), In absolute terms, levels of diagnosed anxiety and depression were highest in the US (38.43% and 46.05%, respectively). Most European countries were generally similar in the prevalence of these comorbidities (anxiety: 23.66% to 36.16%; depression: 24.05% to 39.46%) with the exception of an extremely low prevalence of depression in Italy (7.40%). The prevalence of anxiety and depression were low in both urban China (17.65% and 5.91%, respectively) and Japan (4.17% and 6.12%, respectively).

Pooling the data from the different countries and controlling for the selected list of confounding variables, respondents reporting a diagnosis of HCV reported significantly lower levels of health status summary scores (MCS: −1.87; PCS: −2.51; health utilities: −0.035; all p<.05) compared with those without. Similar effects were observed with health status domain scores, with slightly stronger effects observed in some physical domains (e.g., bodily pain: b = −8.08; physical functioning: b = −6.64) as opposed to psychological ones (e.g., mental health: b = −3.96; emotional role limitations: b = −5.04). Among those employed, significantly greater levels of absenteeism (b = 3.74), presenteeism (b = 7.43), and overall work impairment (b = 8.79) were observed for those reporting a diagnosis of HCV compared with those without (all p<.05). Similarly, across all respondents, increased levels of impairment in daily activities (b = 7.48), more physician visits (b = 2.91) and emergency room visits (b = 0.30) were also observed for those reporting an HCV diagnosis compared with those without. Although a trend was observed for an increased number of hospitalizations (b = 0.28, p = .14), this effect was not significant.

**Figure 1:**
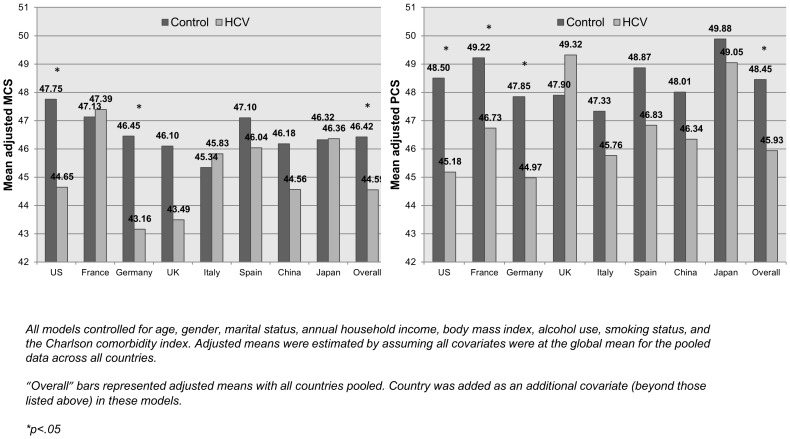
Adjusted mean levels of health status summary scores by HCV diagnosis within each country. *Source: National Health and Wellness Survey.*

The survey data were analyzed for each country separately and the health status burden of HCV across countries compared (see **Figure 1**; pooled estimates are also provided in each figure). The presence of an HCV diagnosis had the greatest effect on MCS in the US (b = −3.11; Adjusted means = 44.65 vs. 47.75), Germany (b = −3.29; Adjusted means = 43.16 vs. 46.45), and the UK (b = −2.61; Adjusted means = 43.49 vs. 46.10). Effects on MCS were minimal and non-significant in France, Italy, Spain, China, and Japan. Effects on PCS were strongest in the US (b = −3.32; Adjusted means = 45.18 VS. 48.50), France (b = −2.49; Adjusted means = 46.73 vs. 49.22), and Germany (b = −2.87; Adjusted means = 44.97 vs. 47.85). Although not significant due to reduced power, trends for lower PCS were observed in Italy, Spain, China, and Japan. A trend for higher PCS among respondents with HCV was observed in the UK, but was not significant.

**Figure 2:**
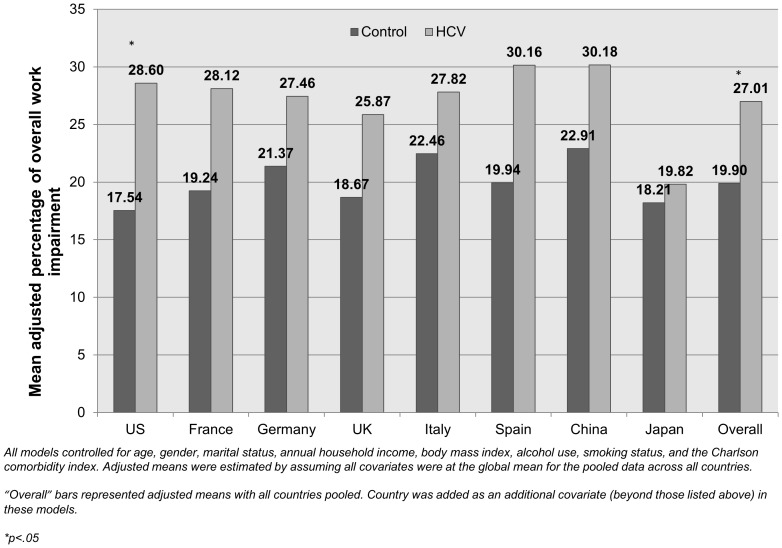
Adjusted mean levels of work productivity and activity impairment by HCV diagnosis within each country. *Source: National Health and Wellness Survey.*

Among those employed, the effects of HCV diagnosis on absenteeism were strongest in the US (b = 4.99) and urban China (b = 4.35) and generally minimal (<3% more than non-HCV controls) elsewhere. Although the effect in France was large (b = 9.70), this value was not significant. The effects of HCV on presenteeism were highest in Spain (b = 18.74) and the US (b = 9.08). Combining absenteeism and presenteeism effects (i.e., overall work impairment), only the differences in the US were significant (Adjusted means = 28.60% vs. 17.54%), however, for all countries other than Japan, respondents with HCV reported 20% more work impairment (see **Figure 2**). Among all, not just those employed, the effects of HCV diagnosis on activity limitations were strongest in the US (b = 11.78), Germany (b = 7.86), and urban China (b = 8.36). Minimal effects on work or activity impairment were observed in Japan.

**Figure 3:**
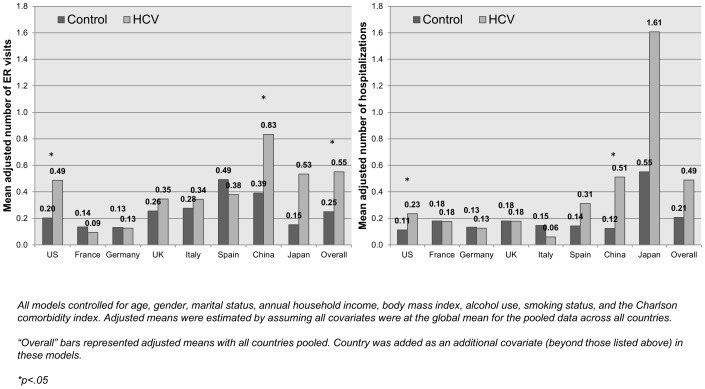
Adjusted mean levels of emergency room visits and hospitalizations by HCV diagnosis within each country. *Source: National Health and Wellness Survey.*

Despite the differences in healthcare systems, the effect of HCV diagnosis on the number of physician visits was consistent across the US, France, Italy, the UK, and urban China (b’s = 1.71 to 2.58). These effects were much larger in Germany (b = 5.05) and Japan (b = 5.18) and minimal in Spain (b = 0.21). Incremental ER visits and hospitalizations among those reporting an HCV diagnosis were observed in the US (b = 0.28 and 0.12, respectively) and urban China (b = 0.44, 0.39) (see **Figure 3**). Although sizeable effects were observed in Japan, these differences were not significant.

## Discussion

Prior research across a number of geographies has suggested a significant burden among those with HCV with respect to health status, work and activity-related impairments, and healthcare resource utilization. However, no study to date has examined these issues across countries using a standardized methodology. Through this approach, comparisons can be made to better understand differences in the characteristics of respondents diagnosed with HCV along with their unmet needs. As many countries lack sufficient ability to monitor HCV trends, such research will be valuable to inform policy-level decisions. The World Health Organization has specifically stated that the lack of current data on HCV epidemiology and burden of disease poses a significant hurdle to healthcare policy-makers and decision-makers [19]. The objective of this study was to help address this gap.

Although providing estimates of the prevalence of HCV was not the sole objective of this study (given its reliance on a patient-reported data source), we did find general consistency with the literature when factoring in diagnosis rate, which is the most relevant comparison [25]–[26]. Our prevalence estimates were slightly higher than the literature for European countries and the United States (possibly due to increased awareness over time or merely sampling error), yet were relationally consistent. For example, within Europe, both Italy and Spain reported the highest rates of HCV which was also found in Cornberg et al (2011) [25]. Fewer studies have examined the prevalence of HCV (factoring in diagnosis rate) in Japan and China making comparisons with our data difficult. Nevertheless, as would be expected, our prevalence figures for reporting an HCV diagnosis is lower than the anti-HCV prevalence in these countries, the latter of which would include those who have not been diagnosed [27].

Naturally, actual population levels of infection are underestimated with a patient-reported approach (as used in our study) as most patients are unaware that they are infected [26]–[28]. Yet, this patient perspective does provide value by helping to document the gap in awareness of HCV infection. This level of underestimation is likely different across countries. Although more research would be necessary to test this hypothesis, it is possible that developing countries (even within urban areas) may be more likely to have a gap between actual disease prevalence and awareness of infection due to reduced healthcare access. This was supported by the fact that patients with HCV in urban China were young, nearly all employed, and affluent which suggests that patients with greater healthcare access in urban China are more likely to be aware of their infection.

Examining the gap between the prevalence of infection and prevalence of awareness can also help to inform public health policy. For example, although most respondents in each country were male, this was not the case in France. Prior research has suggested that the anti-HCV prevalence is higher among women in France, particularly among those middle aged [28]. Nevertheless, our study had an even more skewed distribution. Because we focused on those who are aware of their diagnosis, it is possible that women in France are disproportionately more likely than women in other countries to receive/be aware of a diagnosis or men in France are disproportionately less likely than men in other countries to receive/be aware of a diagnosis. Comparing and contrasting HCV prevalence data from sera and patient self-report can inform where the greatest gaps in HCV awareness may lie, which can identify geographies or subgroups most in the need of patient education.

The age pattern also varied as would be expected given the epidemiological history of HCV. As discussed elsewhere, contaminated needles in the 1930s led to large surge in HCV infection in Japan, resulting in an older respondent population [25]. For a period in Italy before the 1970s, many medical treatments were injected (with needles shared), which also led to a surge in HCV infection and resulting in an older respondent population [26]. The younger HCV population in urban China, along with the low estimated prevalence and high income among respondents, suggests a significant undiagnosed population. It is likely that only those with sufficient healthcare access are being diagnosed.

The comorbidity profile also varied substantially. Given the older age of respondents in Japan, it is not surprising that a high comorbidity burden was also present. Similarly, given the young age of respondents in urban China, it is not surprising that a low burden was observed. However, age and comorbidity burden did not strictly covary. The relatively young respondents of the UK were the most burdened with comorbidities, which can significantly complicate disease management. Interestingly, the older respondents of France, Germany and Italy had fewer additional medical complications. Cultural factors likely led to an underreporting of anxiety and depression in both urban China and Japan. Nevertheless, our results suggest that these psychiatric comorbidities may be relevant during the management of disease, particularly in the US but also in Western Europe.

With all countries pooled, for respondents reporting a diagnosis of HCV, a significant burden was observed on health status, work and activity impairments, and healthcare resource use. Although low sample sizes were present in many countries, country-level analyses did provide some preliminary insight into the different manifestations of respondent burden. The health status burden was strongest in the US, France, and Germany and more modest in other parts of the world. Interestingly, although not significant, respondents in the UK reported better health status after adjusting for sociodemographics and comorbidities. It is possible that because of the substantial comorbidity burden these patients report (the highest of all countries) the marginal effect of HCV on physical health is masked. The strongest effects on work productivity loss were observed in the US, urban China, and in Spain highlighting the increased importance of indirect costs in these countries relative to others when quantifying the overall burden of disease. Direct costs effects (as reported directly from the patient) were the largest in the US, urban China, Germany, and Japan.

### Limitations

It should be emphasized that only respondents who were aware of their diagnosis were included in the HCV group in the analyses. Those infected and unaware would be included in the control group. It is also likely that disenfranchised groups were underrepresented from the NHWS and the full extent of the burden of HCV may have been underestimated. It should also be noted that all demographics, health characteristics, comorbidities, and health outcomes were all provided by the respondent without any objective confirmation of the responses.

## Conclusions

Overall, the patient characteristics and health outcomes of patients diagnosed with HCV can vary significantly by geography, highlighting the unique needs of these patients on a country and regional level. This information is important to ensure proper management is available to these patients. Despite the cross-country differences, however, significant medical unmet needs still exist for patients diagnosed with HCV in the US, Western Europe, and Asia.
